# Ischemic Stroke During Rivaroxaban Therapy: The Importance of Food-Dependent Absorption and Careful Assessment Before Antithrombotic Escalation

**DOI:** 10.7759/cureus.116183

**Published:** 2026-09-13

**Authors:** Mihail Petrov, Teodora Sakelarova, Miroslav Nalbantov, Nikolay Velinov, Nikolay Gabrovsky

**Affiliations:** 1 Neurosurgery, University Multiprofile Hospital for Active Treatment with Emergency Medicine N. I. Pirogov, Sofia, BGR; 2 Neurology, University Multiprofile Hospital for Active Treatment with Emergency Medicine N. I. Pirogov, Sofia, BGR; 3 Cardiology, University Multiprofile Hospital for Active Treatment with Emergency Medicine N. I. Pirogov, Sofia, BGR; 4 Neurological Surgery, University Multiprofile Hospital for Active Treatment with Emergency Medicine N. I. Pirogov, Sofia, BGR

**Keywords:** direct oral anticoagulant (doac), ischemic cerebrovascular disease, stroke, stroke management, therapeutic anticoagulation

## Abstract

Ischemic stroke occurring during direct oral anticoagulant (DOAC) therapy for atrial fibrillation represents a frequent and challenging clinical dilemma due to increasing stroke rates and increasing DOAC prescription rates. Such events are often interpreted as treatment failure, prompting escalation with antiplatelet therapy, particularly in the presence of concomitant intracranial atherosclerotic disease (ICAD), and thereby exposing patients to increased bleeding risk. However, apparent DOAC failure may reflect correctable pharmacokinetic or adherence-related factors.

We report a 75-year-old man with nonvalvular atrial fibrillation who presented with acute left-sided cerebellar ataxia and inability to sit unsupported. Neuroimaging demonstrated a posterior circulation ischemic stroke associated with intracranial atherosclerotic occlusion of the left vertebral artery, with collateral filling of the left posterior inferior cerebellar artery from the contralateral vertebral artery. Endovascular recanalization was unsuccessful, and the patient received short-term tirofiban due to the identified ICAD, with no hemorrhagic transformation on follow-up imaging. Although the stroke occurred during rivaroxaban therapy, detailed history revealed intake of rivaroxaban 20 mg once daily without food, likely resulting in reduced bioavailability.

The event was interpreted as pharmacokinetic underexposure rather than true anticoagulant failure. Antiplatelet escalation was avoided, and anticoagulation was resumed with a food-independent DOAC. The patient remained clinically stable without hemorrhagic complications.

This case highlights the importance of careful medication history and pharmacokinetic awareness in patients presenting with ischemic stroke during DOAC therapy. Recognizing apparent rather than true treatment failure can prevent unnecessary antithrombotic escalation and reduce avoidable bleeding risk.

## Introduction

Direct oral anticoagulants (DOACs) have become the cornerstone of stroke prevention in patients with nonvalvular atrial fibrillation, owing to their favorable balance between efficacy and safety compared with vitamin K antagonists [1,2]. Nevertheless, ischemic stroke continues to occur in a subset of patients receiving DOAC therapy, around 1-2% [3]. This creates a frequent and clinically challenging dilemma in acute stroke care [4].

When stroke occurs during ongoing anticoagulation, the event is often interpreted as treatment failure. This perception may prompt either change of the anticoagulant regimen or escalation of antithrombotic therapy, most commonly through the addition of antiplatelet agents. Such escalation is particularly tempting in the presence of concomitant intracranial atherosclerotic disease (ICAD), where clinicians may seek to address both cardioembolic and large-artery mechanisms simultaneously. However, combined anticoagulant and antiplatelet therapy is associated with a substantially increased risk of hemorrhagic complications, while evidence supporting its routine use after ischemic stroke in patients with atrial fibrillation remains limited [5,6].

Importantly, not all strokes occurring during DOAC therapy represent true anticoagulant failure. In real-world practice, apparent treatment failure may result from modifiable factors such as incorrect dosing, drug-drug interactions, impaired absorption, or non-adherence [7]. Among DOACs, rivaroxaban is unique in that therapeutic doses require intake with food to ensure adequate bioavailability - a detail that may be overlooked in routine clinical assessment, particularly in the acute stroke setting [7].

We present a posterior circulation stroke case in which apparent DOAC failure, combined with intracranial atherosclerotic disease, created strong pressure toward antithrombotic escalation. Careful reassessment of medication intake revealed pharmacokinetic underexposure rather than true anticoagulant failure, allowing avoidance of unnecessary escalation and associated bleeding risk. This case illustrates the importance of systematic medication review and pharmacokinetic awareness in antithrombotic decision-making after ischemic stroke.

## Case presentation

A 75-year-old man with a known history of nonvalvular atrial fibrillation presented with acute onset of left-sided cerebellar ataxia, limb dysmetria, and inability to sit unsupported. His medical history was notable for arterial hypertension, congestive heart failure, diabetes mellitus, and obesity. There was no loss of consciousness. Prior to admission, the patient was functionally independent. He had been receiving oral anticoagulation with rivaroxaban 20 mg once daily. On admission, the National Institutes of Health Stroke Scale (NIHSS) score was 5 [8]. The patient had taken his anticoagulant therapy less than 12 hours before admission on an empty stomach.

Symptom onset occurred the day before presentation; however, the patient did not initially seek medical attention and arrived at the hospital approximately 16 hours after symptom onset.

Initial non-contrast cranial computed tomography (CT) scan demonstrated no acute hemorrhage (Figure 1). Unfortunately, at this point at our center, it is not possible to provide 24/7 MRI coverage for stroke patients.

**Figure 1:**
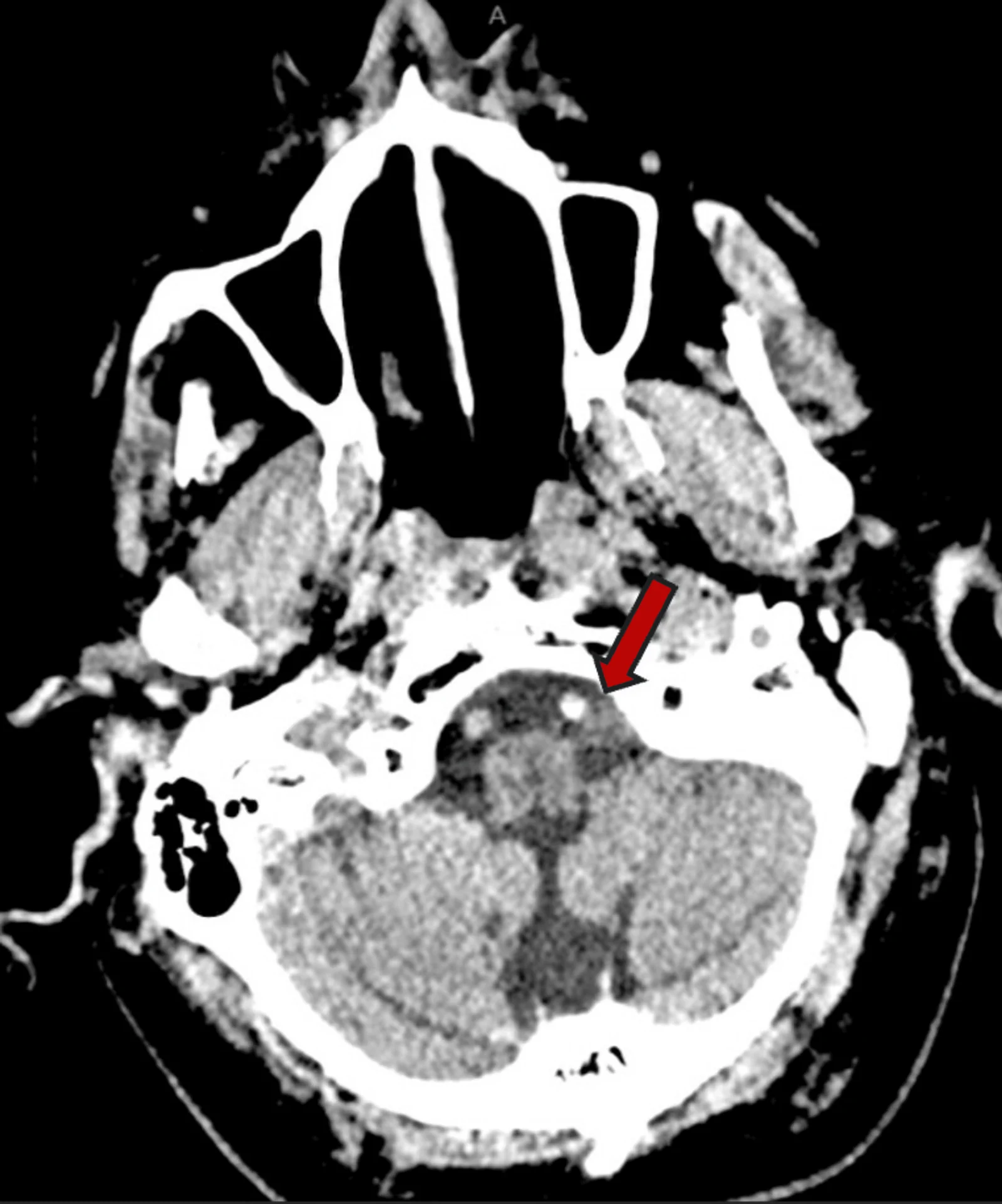
Noncontrast CT scan of the patient at admission, almost 16 hours after symptom onset No apparent early ischemic changes in the left cerebellar hemisphere. A red arrow indicates atherosclerotic calcified changes of the left vertebral artery.

CT angiography (CTA) revealed occlusion of the V4 segment of the left vertebral artery (VA) in the setting of intracranial atherosclerotic disease (Figures 2, 3).

**Figure 2:**
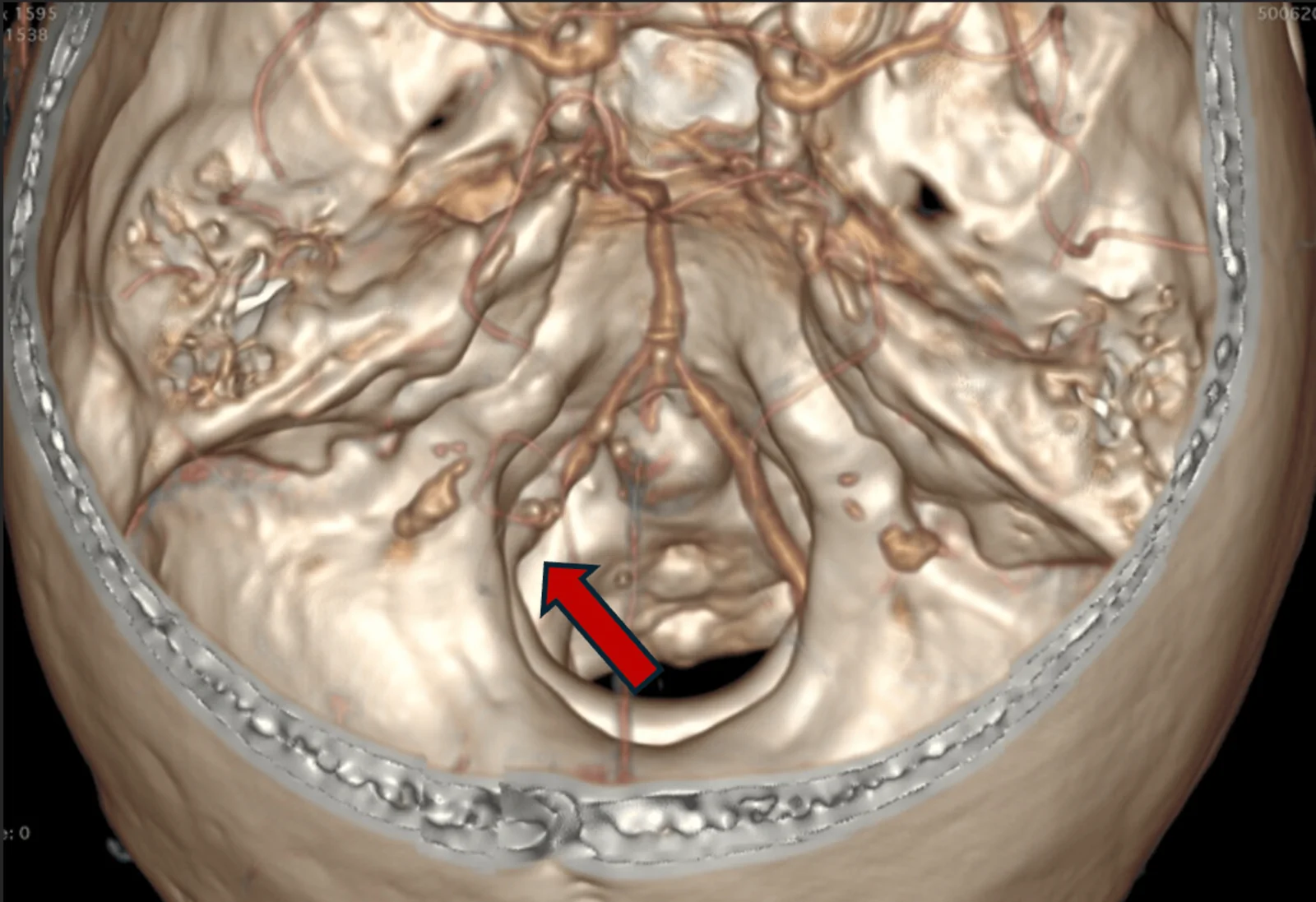
3D reconstruction of the CT-angiography of the patient at admission. A red arrow marks the occlusion of the left VA with irregular contours, signifying probable atherosclerotic plaque. The apparent retrograde filling of the left VA from the right is also visible.

**Figure 3:**
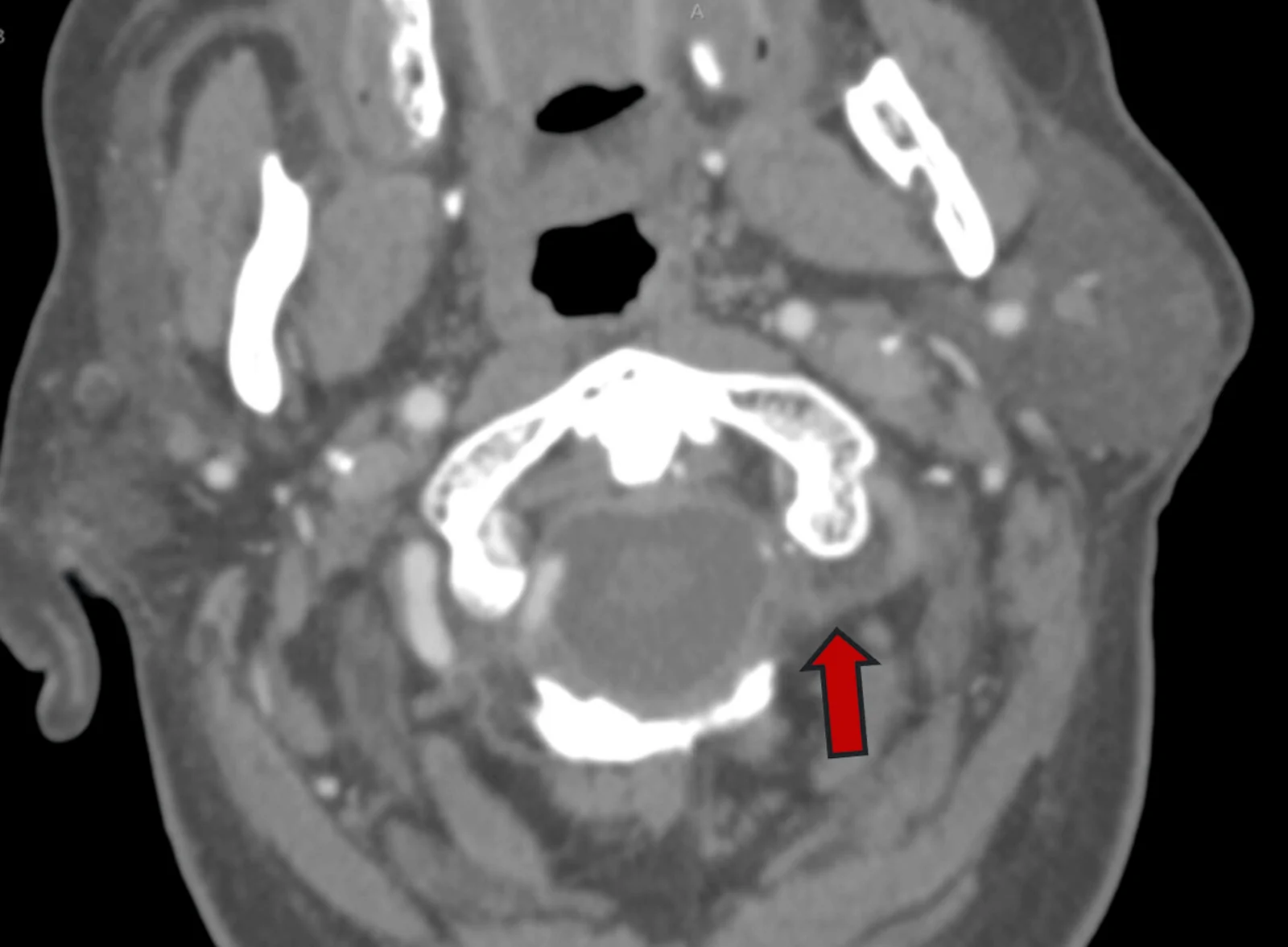
CT angiography of the patient at admission - axial image at the level of C1 vertebrae. A red arrow marks the occlusion of the V3-V4 segment of the left VA. There is still some filling of the left VA, but dramatically less than on the right. The two calcifications medial to the left massa lateralis of the atlas is probably the beginning of the underlying atherosclerotic plaque.

The left posterior inferior cerebellar artery (PICA) was opacified via collateral flow from the contralateral vertebral artery (Figure 4).

**Figure 4:**
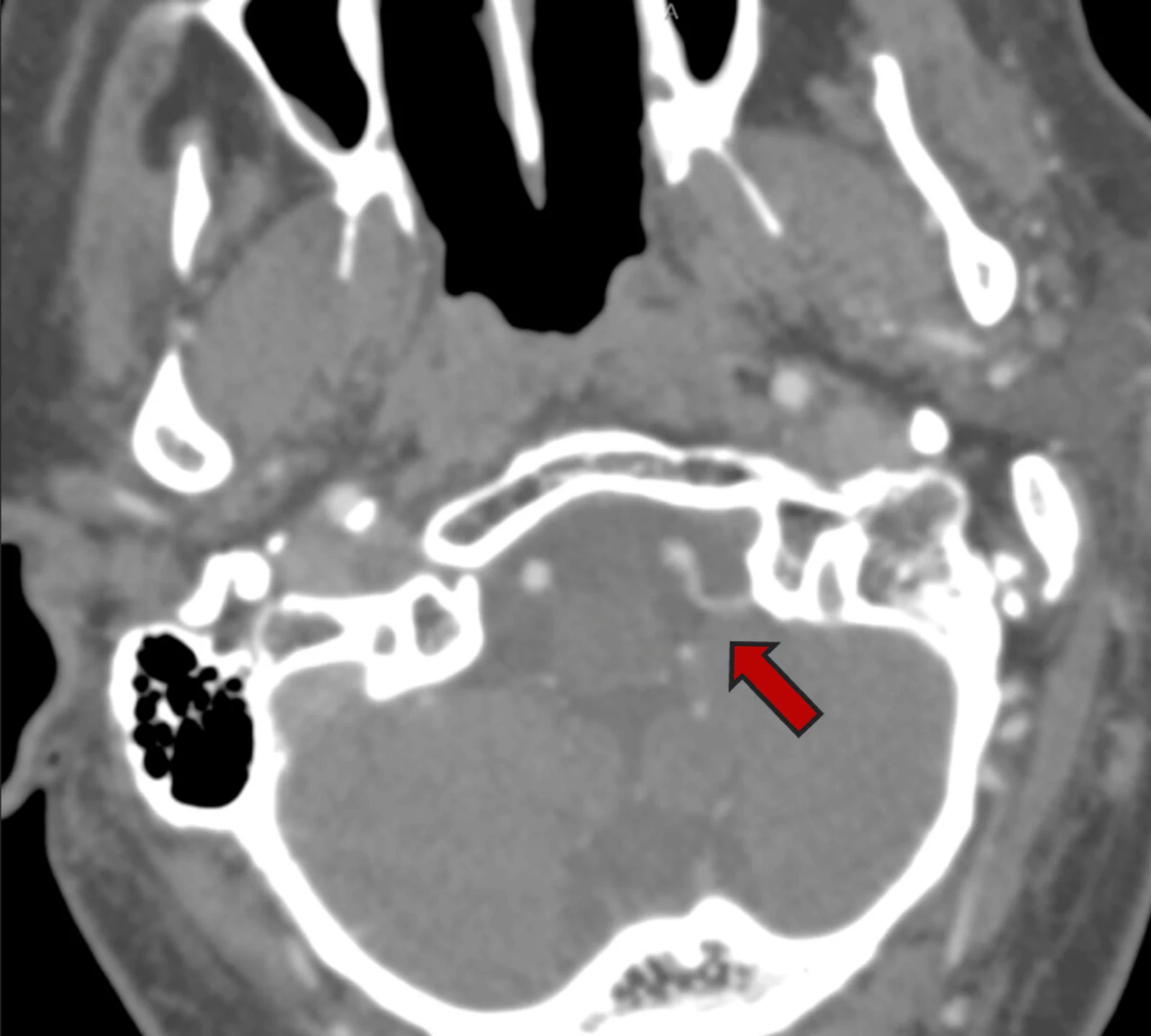
CT angiography of the patient at admission - axial image at the level of foramen magnum. A red arrow marks the retrograde filling of the left VA from the right VA, and the left PICA is visible near the left foramen of Luschka.

These findings were consistent with an acute posterior circulation ischemic stroke.

Given the clinical presentation and imaging findings, endovascular treatment was attempted due to the uncertainty of the underlying pathology of the left VA and the possibility of propagating the thrombus to the basilar artery. A right common femoral artery approach was initiated using an 8-Fr femoral sheath. However, catheterization of the left vertebral artery with an 8-Fr guide catheter (Cerebase guide catheter, Cerenovus, Johnson & Johnson MedTech, Irvine, CA, USA) was unsuccessful despite multiple techniques and diagnostic catheter exchanges. This was due to pronounced tortuosity at the origin of the left subclavian artery and kinking of the V1-V2 segments of the left vertebral artery. The approach was therefore converted to a left radial access using a short 7-Fr radial sheath. A 7-Fr guide catheter (RIST) was successfully positioned at the proximal V3 segment of the left vertebral artery, confirming occlusion of the V4 segment. Three aspiration passes using the ADAPT (A Direct Aspiration First Pass Technique) technique were performed with a 5-Fr aspiration catheter (Sofia aspiration catheter, Terumo Neuro/MicroVention, Aliso Viejo, CA, USA) connected to a Penumbra aspiration pump (Penumbra, Inc., Alameda, CA, USA). Further attempts to cross the occluded segment with a microwire and microcatheter were unsuccessful. Recanalization could not be achieved, with a final Thrombolysis in Cerebral Infarction (TICI) score of 1 [9]. In the context of the identified intracranial atherosclerotic disease, short-term intravenous tirofiban was administered for 24 hours. Follow-up cranial CT performed after 24 hours demonstrated no hemorrhagic transformation (Figure 5). After 24 hours, the patient had left-sided cerebellar ataxia, but he was able to walk with assistance. Rehabilitation was performed during the hospital stay, leading to NIHSS score of 0 at discharge.

**Figure 5:**
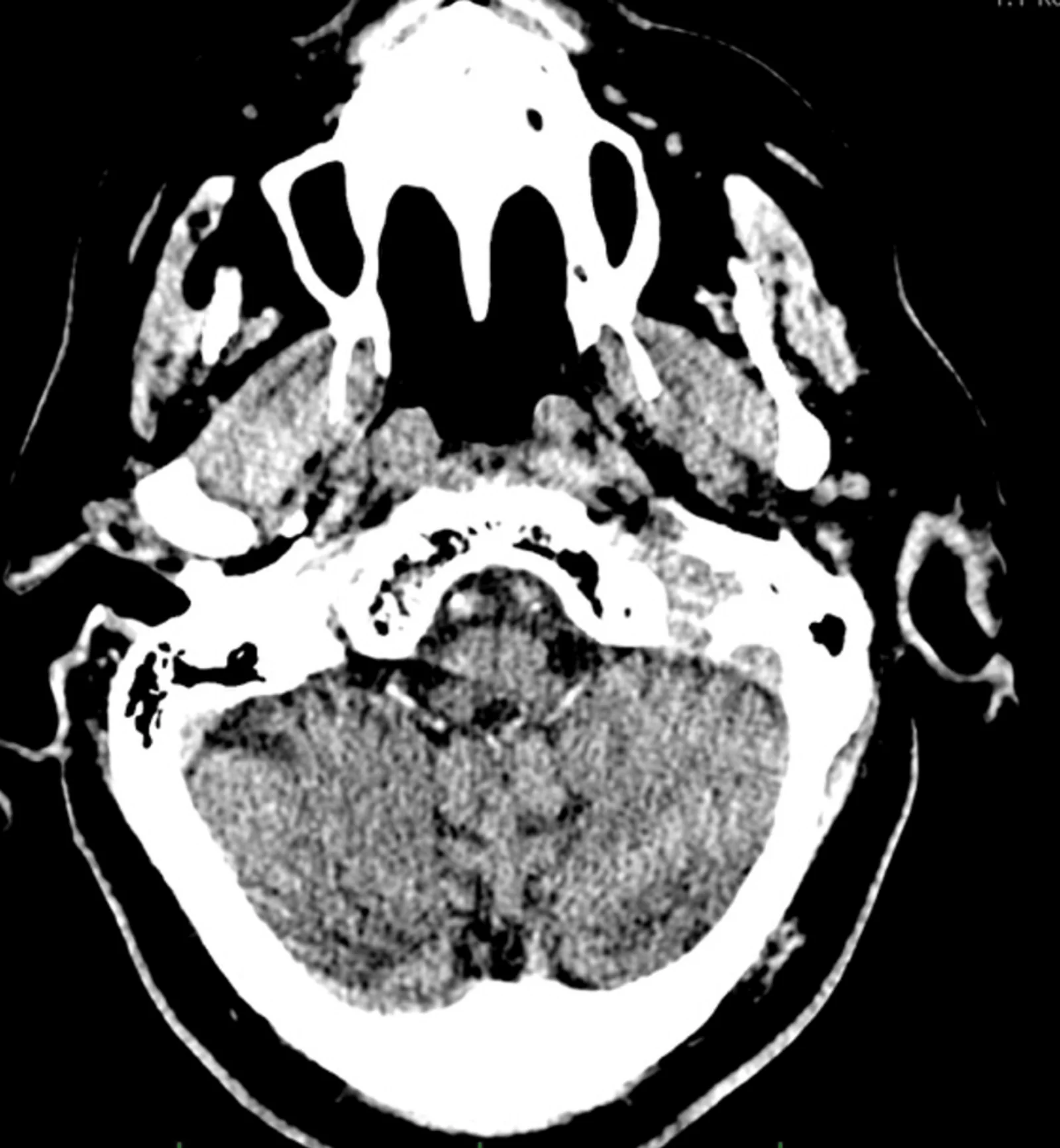
Post-procedure noncontrast CT scan 24 hours after the procedure. No ischemic or hemorrhagic lesions are visible in the left cerebellar hemisphere.

During reassessment of the patient’s medication history, it was noted that although rivaroxaban had been prescribed at the standard dose of 20 mg once daily, the patient reported taking the medication consistently without food, which was also confirmed by his wife and attributed to the lack of information about the medication. No other potential causes of reduced anticoagulant effect, including incorrect dosing, renal impairment, or relevant drug-drug interactions, were identified.

Based on the absence of hemorrhagic complications and the limited infarct burden, the event was interpreted as apparent anticoagulant failure related to pharmacokinetic underexposure rather than true treatment failure. Escalation with antiplatelet therapy was avoided. Anticoagulation was resumed with a food-independent direct oral anticoagulant, Apixaban, in order to improve the patient's compliance. The patient remained clinically stable during hospitalization, with no hemorrhagic or recurrent ischemic events observed. The patient was referred for an echocardiogram on an outpatient basis, which came back unremarkable. The patient was discharged with NIHSS 0 and Modified Rankin Scale (mRS) 1, and at the 90-day follow-up, the patient was mRS 0 [10].

## Discussion

Ischemic stroke occurring despite ongoing direct oral anticoagulant (DOAC) therapy for atrial fibrillation represents a frequent and clinically challenging scenario in contemporary stroke care. Such cases have been increasingly reported in real-world cohorts, highlighting that DOAC therapy, while effective, does not eliminate stroke risk entirely [4]. These events immediately raise concerns regarding treatment failure and secondary prevention adequacy, particularly when recanalization is unsuccessful and imaging demonstrates concomitant ICAD.

The intuitive response in this context is frequently escalation of antithrombotic therapy, most commonly by adding antiplatelet agents to existing anticoagulation. This inclination is reinforced when a large-artery pathology is identified, as clinicians may attempt to address both cardioembolic and atherosclerotic mechanisms simultaneously. However, combined antithrombotic therapy is associated with a substantially increased risk of hemorrhagic complications, while evidence supporting its routine use after ischemic stroke in patients with atrial fibrillation remains limited [5,6].

A critical first step when evaluating stroke occurring during DOAC therapy is to distinguish true anticoagulant failure from apparent or pseudo-failure. True failure implies an ischemic event despite adequate anticoagulant exposure, correct dosing, and appropriate adherence. In contrast, apparent failure may result from modifiable factors, including non-adherence, incorrect dosing, renal dysfunction, drug-drug interactions, or impaired absorption [4], and several recent meta-analyses have underscored that switching anticoagulant classes after stroke on DOAC does not uniformly improve outcomes, emphasizing careful reassessment instead [11].

Observational data suggest that apparent failure is common in routine clinical practice and may account for a substantial proportion of ischemic strokes occurring during DOAC therapy [4]. Careful reassessment of medication history is therefore essential. In the present case, detailed questioning revealed intake of rivaroxaban without food, supporting the interpretation of pharmacokinetic underexposure rather than true anticoagulant failure.

Among available DOACs, rivaroxaban has a distinct pharmacokinetic profile that therapeutic doses (15 mg and 20 mg) require intake with food to ensure adequate bioavailability. Without food, absorption is significantly reduced (around 30-40%), resulting in lower plasma concentrations and potentially insufficient anticoagulant effect [7]; this remains an active clinical concern, with ongoing research dedicated to mitigating absorption variability [12]. While this requirement is clearly documented in pharmacokinetic studies and prescribing information, it may be underappreciated in routine clinical practice, particularly in the acute stroke setting.

Failure to recognize food-dependent absorption can lead to misclassification of ischemic events as anticoagulant failure, prompting inappropriate escalation of therapy. This case illustrates how pharmacokinetic factors, when overlooked, may directly influence clinical decision-making and expose patients to unnecessary risk.

The coexistence of atrial fibrillation and intracranial atherosclerotic disease creates a particularly strong temptation to escalate antithrombotic therapy. ICAD is commonly associated with artery-to-artery embolism, and antiplatelet therapy is well established in patients without an indication for anticoagulation. However, when atrial fibrillation mandates anticoagulation, the routine addition of antiplatelet agents has not been shown to reduce ischemic events and is consistently associated with increased bleeding risk [5,6], and contemporary analyses confirm that anticoagulation alone remains superior to antiplatelet monotherapy for stroke prevention in high-risk cohorts [13].

In the present case, the presence of vertebral artery ICAD combined with failed endovascular recanalization could have justified escalation with antiplatelet therapy. However, such escalation would not have addressed the underlying issue of pharmacokinetic underexposure and would likely have increased the risk of hemorrhagic complications. Current guideline recommendations caution against routine combination therapy in this setting, supporting a more conservative, individualized approach [6].

Posterior circulation strokes differ from anterior circulation events with respect to collateral pathways, infarct evolution, and hemorrhagic risk. In this patient, collateral filling of the posterior inferior cerebellar artery from the contralateral vertebral artery suggested preserved perfusion and limited infarct burden (Figure 4). The absence of hemorrhagic transformation on follow-up imaging further supported a cautious antithrombotic strategy (Figure 5). These factors reduced the urgency for aggressive escalation and supported early resumption of anticoagulation alone. Incorporating vascular anatomy, collateral status, and infarct extent into post-stroke decision-making is particularly important in posterior circulation events, where clinical severity and imaging findings may not align as clearly as in anterior circulation strokes [4].

Why “more” is not better: restraint as an active decision

A central message of this case is that restraint in antithrombotic therapy should be viewed as an active, evidence-based decision rather than therapeutic inertia. Escalation with antiplatelet therapy in this context would have increased bleeding risk without correcting the underlying cause of the ischemic event [5,6,14]. By identifying pharmacokinetic underexposure and avoiding unnecessary escalation, harm was prevented rather than merely deferred. This perspective reframes secondary prevention as a process of precise problem identification rather than maximal pharmacological intervention. In this sense, “doing less” constituted appropriate, patient-centered care.

Practical implications for stroke clinicians

This case supports a structured approach when ischemic stroke occurs during DOAC therapy: Confirmation of correct dosing and adherence; review of renal function and drug-drug interactions; assessment of pharmacokinetic factors, including food-dependent absorption; consideration of antithrombotic escalation only after these factors have been excluded [4,7].

Such an approach emphasizes multidisciplinary collaboration and may reduce unnecessary bleeding complications while preserving effective secondary prevention, as highlighted in recent comprehensive DOAC management reviews in complex clinical scenarios [15,16].

Limitations

This report describes a single patient. MRI was not performed in the workup of the patient, and pharmacokinetic measurements were not available to directly confirm rivaroxaban underexposure. As with all case reports, conclusions are hypothesis-generating rather than definitive. Nevertheless, the scenario reflects a common and underrecognized issue with direct relevance to everyday stroke practice.

## Conclusions

Ischemic stroke occurring during direct oral anticoagulant therapy should not be automatically interpreted as treatment failure. Careful reassessment of medication intake and pharmacokinetic factors may reveal correctable causes of apparent failure and prevent unnecessary escalation of antithrombotic therapy. This case underscores the importance of individualized, evidence-based decision-making to balance ischemic prevention against bleeding risk after stroke.
